# Administratively Defined Functional Vulnerability and Adverse Short-Term Outcomes in Older Adults Hospitalized with Crohn’s Disease Flares: A Propensity-Matched Multicenter Cohort Study

**DOI:** 10.3390/diseases14070225

**Published:** 2026-06-23

**Authors:** Noor Albusta, Mohamed Abdulla, Ali Bosta, Rehab Almarzooq

**Affiliations:** 1 Department of Internal Medicine, Lahey Hospital and Medical Center, Burlington, MA 01805, USA; 2 Department of Medicine, Royal College of Surgeons in Ireland–Medical University of Bahrain, Adliya P.O. Box 15503, Bahrain; 3 Department of Medicine, Salmaniya Medical Complex, Manama P.O. Box 12, Bahrain; aalbosta@hospitals.gov.bh (A.B.); rmarzooq1@hospitals.gov.bh (R.A.)

**Keywords:** Crohn’s disease, inflammatory bowel disease, frailty, older adults, hospitalization, bowel surgery, sepsis, acute kidney injury, readmission, propensity score matching

## Abstract

Background/Objectives: Functional vulnerability may identify older adults hospitalized with Crohn’s disease flares who are at increased risk for adverse outcomes, but its prognostic significance in this setting remains incompletely defined. We evaluated the association between administratively defined functional vulnerability, identified using administrative diagnostic codes, and short-term clinical outcomes among adults aged ≥65 years hospitalized with Crohn’s disease flares. Methods: We conducted a retrospective cohort study using the TriNetX US Collaborative Research Network through February 2026. Functional vulnerability was identified using ICD-10-CM codes for frailty, sarcopenia, cachexia, abnormal weight loss, muscle weakness, gait/mobility abnormalities, or reduced mobility within 12 months before or during the index hospitalization. Patients coded only for nonspecific weakness or fatigue were excluded from the functional vulnerability cohort. Patients underwent 1:1 propensity score matching using demographic, comorbidity, Crohn’s disease-related, medication, nutritional, and laboratory variables. The primary outcome was 30-day all-cause mortality. Results: Among 18,420 eligible patients, 2846 met criteria for functional vulnerability, and 15,574 did not. After matching, 2720 patients remained in each cohort. Functional vulnerability was associated with higher 30-day mortality (RR 1.61, 95% CI 1.21–2.14), 90-day mortality (RR 1.40, 95% CI 1.14–1.72), bowel surgery (RR 1.29, 95% CI 1.07–1.56), sepsis (RR 1.41, 95% CI 1.18–1.68), acute kidney injury (RR 1.26, 95% CI 1.10–1.44), ICU admission (RR 1.32, 95% CI 1.13–1.55), TPN use (RR 1.47, 95% CI 1.20–1.79), and 90-day readmission (RR 1.17, 95% CI 1.07–1.29). Functionally vulnerable patients also had longer hospital stays (8.9 vs. 6.7 days; mean difference 2.2 days, 95% CI 1.9–2.5). Conclusions: Administratively defined functional vulnerability identified through diagnostic coding was associated with worse short-term outcomes among older adults hospitalized with Crohn’s disease flares. Although functional vulnerability is a recognized predictor of adverse outcomes across hospitalized populations broadly, these findings quantify its prognostic significance specifically in Crohn’s disease flare hospitalizations and suggest that functional vulnerability may identify a high-risk geriatric IBD phenotype that could benefit from early multidisciplinary assessment, nutritional optimization, rehabilitation planning, and post-discharge care coordination.

## 1. Introduction

Crohn’s disease is a chronic, immune-mediated inflammatory bowel disease characterized by relapsing transmural inflammation that can involve any segment of the gastrointestinal tract [1,2]. The disease course is heterogeneous and may lead to strictures, fistulas, abscesses, bowel obstruction, malnutrition, anemia, hospitalization, and surgery [1,2,3]. Contemporary management emphasizes objective assessment of inflammation, risk stratification, steroid-sparing therapy, prevention of cumulative bowel damage, and treat-to-target strategies [3,4].

The population of older adults living with inflammatory bowel disease (IBD) continues to grow because of population aging, improved survival, and incident diagnoses in later life [5,6,7,8]. Older adults with IBD are clinically heterogeneous: some remain functionally independent, whereas others have multimorbidity, polypharmacy, malnutrition, sarcopenia, cognitive impairment, infection susceptibility, and vulnerability to treatment-related complications [5,6,8,9]. Chronological age alone is therefore an incomplete marker of risk, and expert guidance emphasizes incorporating biological age, comorbidities, function, frailty, and treatment goals into decision-making for older adults with IBD [5,6].

Hospitalization for a Crohn’s disease flare is an important clinical stressor in older adults. Acute flares may be complicated by dehydration, anemia, infection, venous thromboembolism, intestinal obstruction, intra-abdominal abscess, urgent surgery, corticosteroid exposure, functional decline, and readmission [3,4,5,6,10,11]. Standard inpatient risk stratification often focuses on disease phenotype, inflammatory severity, prior surgery, infection, and medication exposure; however, these factors do not fully capture physiologic vulnerability.

Frailty is a multidimensional syndrome of diminished physiologic reserve that increases vulnerability to adverse outcomes after acute illness, hospitalization, or procedural stress [12,13]. The Fried phenotype conceptualizes frailty using physical features such as weakness, weight loss, exhaustion, slowness, and low activity [12], while the Clinical Frailty Scale and Hospital Frailty Risk Score provide clinically and administratively usable approaches to identifying frailty risk [14,15]. Claims-based frailty instruments have also been developed and validated using administrative data, including the Johns Hopkins claims-based frailty indicator and the Kim claims-based frailty index [16,17,18,19].

Functional vulnerability is particularly relevant to IBD because chronic inflammation, malnutrition, sarcopenia, anemia, corticosteroid exposure, infection, and repeated hospitalization may interact with age-related physiologic decline and reduced functional reserve. Prior work in hospitalized IBD populations has shown that frailty and related vulnerability syndromes are associated with mortality, readmission, and greater hospitalization burden. However, the association between functional vulnerability identified through administrative diagnostic codes and short-term outcomes among older adults hospitalized with Crohn’s disease flares remains incompletely characterized in large multicenter real-world cohorts.

To address this gap, we conducted a retrospective cohort study using the TriNetX US Collaborative Research Network. We evaluated whether administratively defined functional vulnerability, identified using diagnostic codes reflecting frailty-related conditions, was associated with mortality, bowel surgery, sepsis, acute kidney injury, intensive care unit admission, total parenteral nutrition use, readmission, and hospital length of stay after propensity score matching for demographic, comorbidity, disease-related, medication, nutritional, and laboratory variables. Throughout this manuscript, the term “functional vulnerability” is used consistently to describe the study exposure—an administratively defined construct based on diagnostic codes for frailty-related conditions—to distinguish it from frailty as measured by validated clinical instruments such as the Fried phenotype or the Clinical Frailty Scale.

## 2. Materials and Methods

### 2.1. Study Design

We performed a retrospective cohort study using the TriNetX US Collaborative Research Network (TriNetX LLC, Cambridge, MA, USA), a federated electronic health record database that aggregates de-identified data from healthcare organizations across the United States [20,21]. Available data include demographics, diagnoses, procedures, medications, laboratory values, vital signs, and healthcare encounters. Cohort identification, data extraction, propensity score matching, and statistical analyses were conducted using the TriNetX Analytics Platform (version 1.0, TriNetX LLC, Cambridge, MA, USA). This study used de-identified, HIPAA-compliant data and therefore did not require institutional review board approval. The study was conducted in accordance with the STROBE guidelines for observational research [22].

Because TriNetX is a federated EHR network, patient-level chart review, endoscopic severity, radiographic findings, operative details, exact inpatient medication timing, validated frailty instruments, and physician global assessment are not uniformly available. Therefore, exposures and outcomes were defined using structured diagnosis, procedure, medication, laboratory, and encounter data.

### 2.2. Study Population and Cohort Definitions

We identified adults aged ≥65 years hospitalized with Crohn’s disease flare through February 2026. Crohn’s disease was identified using ICD-10-CM code K50.x. The index date was defined as the date of the qualifying inpatient encounter.

To improve specificity for flare-related hospitalization, the primary cohort required Crohn’s disease (K50.x) to be coded as the primary diagnosis or encounter-associated diagnosis for the index hospitalization when available in TriNetX. In addition, patients were required to have at least one flare-supporting feature recorded during the index hospitalization or immediately adjacent encounter window, including systemic corticosteroid administration, intestinal obstruction, gastrointestinal bleeding, intra-abdominal abscess, IBD-related endoscopic evaluation, IBD-related abdominal imaging, abdominal pain/diarrhea diagnosis, or elevated inflammatory markers when available. This operational definition was intended to enrich for Crohn’s disease flare admissions while acknowledging that administrative data cannot perfectly distinguish flare-driven hospitalizations from admissions in which Crohn’s disease is a comorbidity diagnosis.

Patients were classified into two cohorts according to the presence or absence of administratively defined functional vulnerability within 12 months before or during the index hospitalization.

Functional vulnerability cohort: Patients with at least one ICD-10-CM code for functional vulnerability, including R54 (age-related physical debility/frailty), M62.84 (sarcopenia), R64 (cachexia), R63.4 (abnormal weight loss), M62.81 (muscle weakness), R26.89 (other abnormalities of gait and mobility), or Z74.09 (other reduced mobility), were assigned to this group. These codes were grouped to approximate an administratively defined functional vulnerability phenotype rather than directly measured physical frailty. The rationale for grouping these constructs is that claims- and EHR-based frailty instruments commonly rely on deficit accumulation across mobility limitation, weight loss, weakness, sarcopenia, disability, and dependence domains rather than on a single frailty diagnosis code [15,16,17,18]. This approach is conceptually related to, but not identical to, validated administrative frailty tools such as the Hospital Frailty Risk Score, the Johns Hopkins claims-based frailty indicator, and the Kim claims-based frailty index, which use broader and differently weighted code sets [15,16,17,18]. Therefore, throughout this manuscript, the exposure is described as administratively defined functional vulnerability rather than physiologically measured frailty.

To improve specificity, patients coded only for nonspecific weakness or fatigue without another qualifying functional vulnerability-related code were excluded from the functional vulnerability cohort. Specifically, isolated R53.1 (weakness) and/or R53.83 (other fatigue) without R54, M62.84, R64, R63.4, M62.81, R26.89, Z74.09, or another mobility/debility code were not considered sufficient for functional vulnerability cohort assignment.

Non-functional vulnerability cohort: Patients hospitalized with Crohn’s disease flare without qualifying functional vulnerability-related codes during the same baseline window were included in this cohort.

Exclusion criteria applied to both cohorts included age <65 years, incomplete demographic data, ulcerative colitis without Crohn’s disease, colorectal or small-bowel malignancy before index hospitalization, elective surgery without flare-related hospitalization, and insufficient baseline data for propensity matching. Patients missing age, sex, or race/ethnicity were excluded. Patients were not excluded solely for missing laboratory or BMI values; missingness handling is described below.

### 2.3. Baseline Characteristics and Covariates

Baseline variables were selected a priori based on clinical relevance and availability in TriNetX. Demographic variables included age, sex, race/ethnicity, and body mass index when available. Comorbidities documented within 12 months before index hospitalization included hypertension, type 2 diabetes, chronic kidney disease, coronary artery disease, heart failure, chronic obstructive pulmonary disease, dementia, depression, osteoporosis, anemia, malnutrition, chronic liver disease, and a history of venous thromboembolism.

Crohn’s disease-related variables included prior bowel resection, prior intestinal obstruction, stricturing phenotype proxy, penetrating phenotype proxy, perianal disease, prior intra-abdominal abscess, prior hospitalization, corticosteroid exposure, biologic exposure, immunomodulator exposure, 5-aminosalicylate exposure, antibiotic exposure, and opioid exposure. Laboratory covariates measured within 90 days before or during the index hospitalization included hemoglobin, albumin, creatinine, white blood cell count, platelet count, and C-reactive protein when available.

Among the 18,420 eligible patients, BMI data were available for 13,248 (71.9%), hemoglobin for 16,762 (91.0%), albumin for 14,106 (76.6%), creatinine for 16,946 (92.0%), white blood cell count for 16,578 (90.0%), platelet count for 16,394 (89.0%), and C-reactive protein for 9948 (54.0%). Missing data proportions were similar between the functional vulnerability and non-functional vulnerability cohorts for most variables, although BMI missingness was modestly higher in the functional vulnerability cohort (31.2% vs. 27.4%). Missing-indicator terms were included in the propensity score model for all covariates with incomplete data, as described below.

### 2.4. Study Endpoints and Outcome Definitions

The primary outcome was 30-day all-cause mortality after index hospitalization.

Secondary outcomes included 7-day and 90-day all-cause mortality; bowel surgery within 30 days, including small-bowel resection, ileocolic resection, colectomy, or ostomy creation; sepsis within 30 days; acute kidney injury within 30 days; intensive care unit admission within 30 days; venous thromboembolism within 30 days; total parenteral nutrition use within 30 days; blood transfusion within 30 days; 30-day and 90-day all-cause readmission; and hospital length of stay.

Outcomes were defined using ICD-10-CM diagnosis codes, ICD-10-PCS procedure codes, CPT codes, medication records, and encounter data available within TriNetX. Crohn’s disease was identified using ICD-10-CM K50.x. Administratively defined functional vulnerability was identified using R54, M62.84, R64, R63.4, M62.81, R26.89, and Z74.09; isolated R53.1 and/or R53.83 without another qualifying functional vulnerability-related code were not considered sufficient for functional vulnerability cohort assignment. Sepsis was identified using ICD-10-CM A40.x, A41.x, R65.2x, and related severe sepsis or septic shock codes. Acute kidney injury was identified using ICD-10-CM N17.x. Venous thromboembolism was identified using ICD-10-CM I26.x, I80.x, I82.x, and related pulmonary embolism or deep venous thrombosis codes. Bowel surgery was identified using ICD-10-PCS and CPT procedure codes for small-bowel resection, ileocolic resection, colectomy, ostomy creation, and related intestinal resection procedures. Blood transfusion was identified using ICD-10-PCS transfusion codes, CPT transfusion codes, or blood product administration records. Intensive care unit admission was identified using ICU encounter/location data when available, or critical care encounter codes such as CPT 99291 and 99292 when location-based data were unavailable. Total parenteral nutrition use was identified using medication or nutrition administration records and corresponding parenteral nutrition procedure or HCPCS codes when available. Readmission was defined as a subsequent inpatient encounter within 30 or 90 days after the index discharge or index encounter date. Mortality was identified using the TriNetX structured mortality status within the specified follow-up window.

### 2.5. Statistical Analysis

Baseline characteristics were compared using chi-square tests for categorical variables and Student’s *t*-tests or Wilcoxon rank-sum tests for continuous variables, as appropriate. We performed 1:1 propensity score matching using nearest-neighbor matching without replacement. A caliper width of 0.2 standard deviations of the logit of the propensity score was used, consistent with conventional recommendations for propensity score matching in observational studies [23].

Covariates included demographics, comorbidities, Crohn’s disease phenotype proxies, prior bowel resection, medication exposures, nutritional markers, laboratory parameters, and missing-indicator terms for covariates with incomplete data. Covariate balance after matching was assessed using standardized mean differences (SMDs), with values <0.10 considered balanced. The C-statistic of the propensity score model was recorded as a measure of discrimination.

For binary outcomes, relative risks (RRs) and risk differences (RDs) with 95% confidence intervals (CIs) were calculated. Continuous outcomes were reported as means with standard deviations and compared using mean differences with 95% CIs. Cox proportional hazards models were performed in the matched cohort for time-to-event outcomes, and hazard ratios (HRs) with 95% CIs were reported. The proportional hazards assumption was assessed using Schoenfeld residuals for all Cox models reported, including 90-day all-cause mortality, bowel surgery, sepsis, acute kidney injury, ICU admission, TPN use, and 90-day readmission. No statistically significant violations of the proportional hazards assumption were identified for any of these models (all global test *p* > 0.05).

Sensitivity analyses included (1) a restrictive functional vulnerability definition requiring at least two qualifying functional vulnerability-related codes within 12 months; (2) exclusion of patients with baseline malnutrition/cachexia codes to assess whether functional vulnerability associations persisted beyond nutritional coding; (3) complete-case analysis among patients with available key laboratory covariates; and (4) subgroup analyses stratified by stricturing/penetrating phenotype proxies, prior bowel resection, and biologic exposure. Subgroup analyses were exploratory, and interaction testing was used to assess heterogeneity.

Statistical significance was defined as a two-sided *p*-value < 0.05. All analyses were conducted within the TriNetX analytics platform.

## 3. Results

### 3.1. Study Population

**Figure 1:**
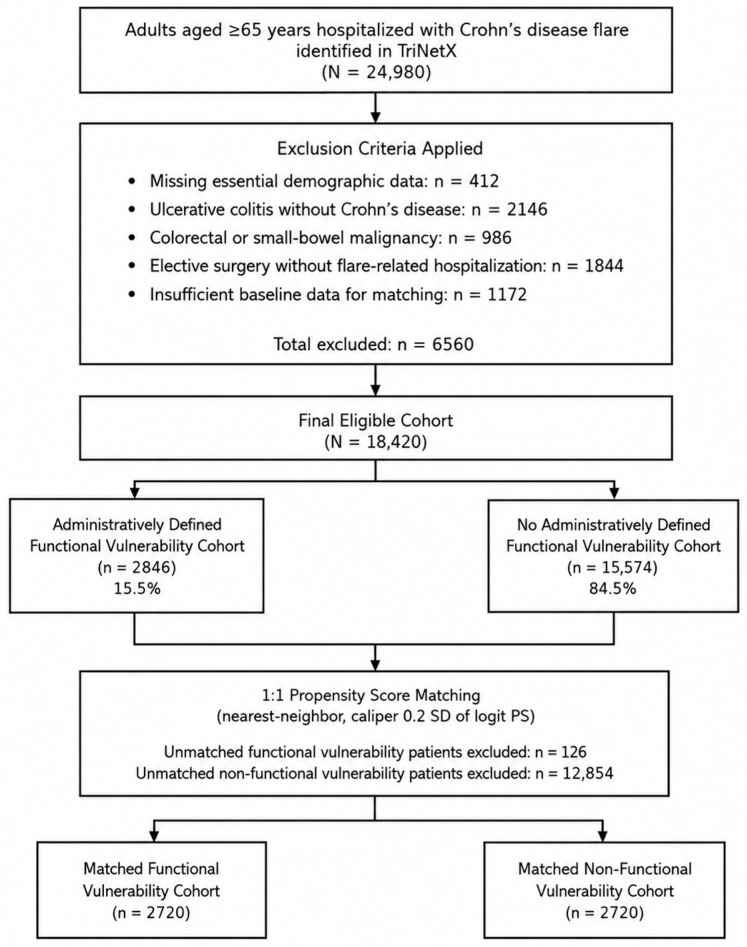
Study flow diagram showing cohort selection, exclusion criteria, propensity score matching, and final matched cohorts.

A total of 24,980 adults aged ≥65 years with Crohn’s disease and qualifying inpatient encounters were identified. After applying exclusion criteria, 18,420 patients comprised the eligible cohort: 2846 patients with administratively defined functional vulnerability and 15,574 without functional vulnerability. After 1:1 propensity score matching, 2720 patients remained in each cohort. The propensity score model demonstrated acceptable discrimination, with a C-statistic of 0.74. After matching, 2720 of 2846 functionally vulnerable patients were retained, corresponding to 95.6% retention of the functional vulnerability cohort; 126 functionally vulnerable patients (4.4%) could not be matched and were excluded from matched analyses. The cohort selection process is summarized in Figure 1.

### 3.2. Baseline Characteristics

Before matching, functionally vulnerable patients were older and had higher prevalences of chronic kidney disease, heart failure, chronic obstructive pulmonary disease, dementia, anemia, malnutrition/cachexia, prior intestinal obstruction, prior bowel resection, and corticosteroid exposure. Functionally vulnerable patients also had lower mean albumin and hemoglobin and higher inflammatory markers. After matching, all measured covariates were balanced with SMDs < 0.10 (Table 1).

### 3.3. Primary and Secondary Outcomes

After matching, functional vulnerability was associated with higher 30-day all-cause mortality. Functional vulnerability was also associated with higher 7-day and 90-day mortality, bowel surgery, sepsis, AKI, ICU admission, VTE, TPN use, blood transfusion, and 30- and 90-day readmission (Table 2).

### 3.4. Continuous Outcomes

Functionally vulnerable patients had a longer hospital length of stay, higher peak creatinine, greater creatinine increase from baseline, lower hemoglobin nadir, and higher peak CRP (Table 3).

### 3.5. Time-to-Event Models

Cox proportional hazards models confirmed higher hazards of mortality, bowel surgery, sepsis, AKI, ICU admission, TPN use, and readmission among functionally vulnerable patients in the matched cohort (Table 4). The proportional hazards assumption was formally assessed using Schoenfeld residuals for all models in Table 4; no statistically significant violations were identified (all global test *p* > 0.05).

### 3.6. Sensitivity Analyses

Sensitivity analyses using a restrictive functional vulnerability definition requiring at least two functional vulnerability-related codes produced directionally consistent findings, with modestly larger effect estimates. Associations also persisted after excluding patients with baseline malnutrition/cachexia codes (Table 5).

### 3.7. Subgroup Analyses

Exploratory subgroup analyses showed directionally consistent associations between functional vulnerability and adverse outcomes across Crohn’s disease phenotype proxies, prior bowel resection status, and biologic exposure. Interaction testing did not demonstrate statistically significant heterogeneity across subgroups (Table 6).

## 4. Discussion

In this large propensity-matched multicenter cohort of older adults hospitalized with Crohn’s disease flares, administratively defined functional vulnerability was associated with worse short-term outcomes across multiple domains. Functionally vulnerable patients had higher risks of mortality, bowel surgery, sepsis, AKI, ICU admission, TPN use, blood transfusion, longer hospital stay, and readmission after matching for demographics, comorbidities, Crohn’s disease phenotype proxies, prior bowel resection, medication exposure, nutritional markers, and laboratory parameters. These findings suggest that functional vulnerability captures clinically meaningful risk not fully explained by age, comorbidity burden, inflammatory markers, or Crohn’s disease phenotype alone.

This study builds on two intersecting bodies of literature: geriatric IBD and administrative functional vulnerability measurement. Older adults with IBD are increasingly common and have unique management challenges related to multimorbidity, polypharmacy, infection risk, treatment tolerability, and functional decline [5,6,7,8,9,24]. At the same time, frailty and related constructs of functional vulnerability have emerged as measures of biologic vulnerability that predict adverse outcomes beyond chronological age and comorbidity burden [12,13,14,15,16,17,18]. Prior hospitalized IBD data have shown that frailty is associated with higher mortality, readmission, and hospitalization burden. Our findings extend this literature by focusing specifically on older adults hospitalized with Crohn’s disease flares and by evaluating a broad set of short-term outcomes after propensity matching.

The administrative functional vulnerability definition used in this study intentionally combined frailty-adjacent domains, including sarcopenia, cachexia, abnormal weight loss, weakness, gait abnormality, and reduced mobility. This approach requires careful interpretation. These constructs are not interchangeable with directly measured frailty, and our code set is not identical to validated administrative frailty tools such as the Hospital Frailty Risk Score, the Johns Hopkins claims-based frailty indicator, or the Kim claims-based frailty index [15,16,17,18]. However, each of these instruments relies on the principle that frailty can be approximated from accumulated deficits across functional, mobility, nutritional, cognitive, and disease domains when direct performance measures are unavailable. Therefore, our exposure should be interpreted as an administratively defined functional vulnerability phenotype rather than a validated bedside frailty diagnosis.

Unlike the Hospital Frailty Risk Score and other claims-based frailty indices, our approach intentionally prioritized a transparent and clinically interpretable group of frailty-related diagnoses. This strategy may have improved specificity for functional vulnerability but could differ in sensitivity and overall operating characteristics compared with validated administrative frailty instruments. Future studies directly comparing these approaches in older adults with IBD are warranted.

It is important to acknowledge that administrative coding for functional vulnerability-related conditions is likely incomplete. Coding practices for diagnoses such as sarcopenia (M62.84), frailty (R54), and reduced mobility (Z74.09) vary substantially across institutions, and these conditions are frequently underrecognized and undercoded in routine clinical practice. This ascertainment bias would be expected to misclassify some truly vulnerable patients into the non-functional vulnerability cohort, thereby biasing the observed associations toward the null. Consequently, the effect estimates reported in this study may represent conservative approximations of the true association between functional vulnerability and adverse outcomes.

Additionally, several of the diagnostic codes used to define functional vulnerability—particularly abnormal weight loss (R63.4), cachexia (R64), and muscle weakness (M62.81)—may reflect active Crohn’s disease severity rather than pre-existing functional vulnerability. Chronic intestinal inflammation, malabsorption, and catabolic stress from active Crohn’s disease can independently produce weight loss, sarcopenia, and weakness, making it difficult to distinguish disease-driven debility from true age-related functional vulnerability using administrative codes alone. Although propensity score matching included inflammatory markers (CRP), nutritional parameters (albumin, BMI), anemia, malnutrition coding, and corticosteroid exposure as proxies for disease severity, and the sensitivity analysis excluding malnutrition/cachexia codes showed persistent associations, residual confounding by unmeasured disease activity cannot be excluded.

The functional vulnerability definition included diagnostic codes recorded within 12 months before or during the index hospitalization. Ideally, distinguishing patients with pre-existing functional vulnerability codes from those in whom these codes first appeared during the acute admission would strengthen causal interpretation, as pre-existing codes would more likely reflect baseline vulnerability, whereas codes appearing only during hospitalization may reflect acute illness severity. However, the TriNetX platform does not reliably distinguish the precise timing of diagnosis code entry relative to the admission date at the encounter level, precluding this subanalysis. Future studies using data sources that permit temporal separation of pre-admission and inpatient diagnoses should evaluate whether the prognostic significance of functional vulnerability differs by timing of code ascertainment.

The observed association between functional vulnerability and mortality is clinically plausible. In Crohn’s disease flare admissions, physiologic stress may arise from systemic inflammation, dehydration, anemia, infection, intestinal obstruction, corticosteroid exposure, malnutrition, urgent surgery, or complications of immunosuppressive therapy [1,2,3,4,5,6,19]. Rather than acting through a single pathway, functional vulnerability likely integrates multiple vulnerability domains, explaining its association with diverse outcomes, including mortality, sepsis, AKI, ICU admission, TPN use, and readmission.

The association with bowel surgery is also important. Crohn’s disease can progress to stricturing and penetrating complications requiring operative intervention [1,2,3,4]. Functionally vulnerable patients may have higher cumulative disease burden, poorer nutritional reserve, delayed presentation, more severe obstruction, or reduced ability to tolerate prolonged medical rescue therapy. Although matching included prior bowel resection and phenotype proxies, administrative data do not capture stricture length, fistula anatomy, abscess size, endoscopic severity, radiographic severity, or surgical urgency. Therefore, the observed association should not be interpreted as functional vulnerability causing surgery, but rather as functional vulnerability identifying a higher-risk hospitalized phenotype in which early multidisciplinary planning may be beneficial.

The increased risks of sepsis, AKI, ICU admission, and VTE support the need for functional vulnerability-aware inpatient care. Older functionally vulnerable patients may be more vulnerable to infection because of immunosenescence, malnutrition, corticosteroid exposure, biologic or immunomodulator therapy, healthcare exposure, and indwelling devices. AKI may occur through dehydration, sepsis-associated hypoperfusion, nephrotoxic exposure, contrast administration, or hemodynamic instability. VTE risk is also clinically relevant in IBD hospitalizations, and consensus statements emphasize prevention and treatment of thrombotic events in IBD. These findings support early attention to volume status, infection evaluation, medication reconciliation, renal protection, VTE prophylaxis, and nutritional support.

The association between functional vulnerability and readmission has practical implications for discharge planning. Readmissions after Crohn’s disease flare may reflect recurrent inflammation, obstruction, dehydration, infection, medication adverse events, malnutrition, or gaps in outpatient access. Functional vulnerability may identify patients who require earlier post-discharge follow-up, clearer escalation plans, medication reconciliation, home services, rehabilitation, nutrition support, and coordination between gastroenterology and primary care.

It should be acknowledged that frailty and functional vulnerability are well-established predictors of adverse outcomes across diverse hospitalized populations, including surgical, cardiac, oncologic, and general medical cohorts. The present findings may therefore reflect, at least in part, the general prognostic impact of diminished physiologic reserve in hospitalized older adults rather than a Crohn’s disease-specific phenomenon. This study cannot determine whether the observed associations are unique to Crohn’s disease flare hospitalizations. However, the value of these findings lies in quantifying the magnitude of these associations specifically within the geriatric Crohn’s disease population, demonstrating that the associations persist after matching for Crohn’s-specific variables, including disease phenotype, prior bowel resection, biologic exposure, and inflammatory markers, and identifying a practical, administratively identifiable high-risk phenotype that could trigger disease-specific interventions such as nutritional optimization for IBD-related malnutrition, steroid-sparing strategies, surgical prehabilitation, and coordinated post-discharge gastroenterology follow-up.

Sensitivity analyses strengthened the interpretation of the findings. A stricter ≥2-code functional vulnerability definition showed directionally consistent and slightly larger associations, suggesting that more specific administrative functional vulnerability ascertainment identifies an even higher-risk phenotype. Associations also persisted after excluding malnutrition/cachexia codes, supporting the concept that functional vulnerability-associated risk extends beyond nutritional coding alone. Subgroup analyses showed directionally consistent findings across stricturing/penetrating phenotype proxies, prior bowel resection, and biologic exposure, although these exploratory analyses were not powered to prove subgroup-specific differences.

Nutritional and inflammatory variables, including albumin, hemoglobin, anemia, malnutrition, and inflammatory markers, were included in the propensity score model because they are important determinants of hospitalization outcomes and potential confounders of the relationship between functional vulnerability-related coding and adverse events. However, some of these variables may also partially lie along the causal pathway linking functional vulnerability and poor outcomes. Consequently, the observed associations may be conservative and potentially reflect partial overadjustment.

Several limitations warrant emphasis. First, administratively defined functional vulnerability may misclassify patients because it relies on structured codes rather than validated instruments such as the Fried phenotype, Clinical Frailty Scale, gait speed, grip strength, or comprehensive geriatric assessment. In addition, coding practices vary across institutions, and functional vulnerability-related conditions may be underreported. Second, our composite functional vulnerability definition includes related but distinct constructs, including sarcopenia, cachexia, weight loss, gait abnormality, and reduced mobility. Although this approach is consistent with the deficit-accumulation logic used in administrative frailty research, it is not equivalent to established claims-based tools and should be interpreted cautiously [15,16,17,18]. Some included diagnoses may also reflect active Crohn’s disease severity, malnutrition, or acute illness rather than functional vulnerability alone. Third, the definition of Crohn’s disease flare hospitalization may misclassify some index events because administrative data cannot fully distinguish primary flare admissions from hospitalizations where Crohn’s disease is coded as a comorbidity. To reduce this risk, the primary analysis required Crohn’s disease to be coded as a primary or encounter-associated diagnosis when available and required flare-supporting features, but residual misclassification remains possible. Fourth, missingness in the TriNetX laboratory and BMI variables may introduce bias. Among the 18,420 eligible patients, BMI was available for 71.9%, hemoglobin for 91.0%, albumin for 76.6%, creatinine for 92.0%, white blood cell count for 90.0%, platelet count for 89.0%, and C-reactive protein for 54.0%. Missing data proportions were similar between cohorts for most variables, although BMI missingness was modestly higher in the functional vulnerability cohort (31.2% vs. 27.4%). The primary analysis used missing-indicator handling rather than complete-case exclusion, but missingness itself may reflect differences in care patterns or disease severity. Fifth, despite propensity score matching, residual confounding by unmeasured disease severity remains possible because TriNetX does not uniformly capture endoscopic severity, Harvey–Bradshaw Index, Crohn’s Disease Activity Index, stricture length, abscess size, fistula anatomy, operative urgency, steroid dose/duration, frailty instrument scores, social support, or functional status. In addition, some matched variables, including nutritional and inflammatory markers, may partially overlap with manifestations of functional vulnerability, raising the possibility of overadjustment. Sixth, outcomes defined through structured codes may be misclassified, and mortality/readmission may be incompletely captured if patients receive care outside participating healthcare organizations. Seventh, multiple secondary outcomes were evaluated without adjustment for multiple comparisons; therefore, secondary findings should be interpreted as supportive and exploratory. Finally, this observational study demonstrates association rather than causation.

## 5. Conclusions

In this large multicenter propensity-matched cohort, administratively defined functional vulnerability was associated with worse short-term outcomes among older adults hospitalized with Crohn’s disease flares, including higher mortality, bowel surgery, sepsis, AKI, ICU admission, TPN use, longer hospital stay, and readmission. Functional vulnerability may identify a high-risk geriatric IBD phenotype and should prompt early multidisciplinary assessment, nutritional optimization, careful medication selection, rehabilitation planning, and post-discharge care coordination. Prospective studies using validated frailty instruments and granular Crohn’s disease activity measures are needed to confirm these findings and determine whether functional vulnerability-targeted interventions can improve outcomes.

## Figures and Tables

**Table 1:** Baseline demographic and clinical characteristics before and after propensity score matching.

Characteristic	Before PSM Functional Vulnerability (N = 2846)	Before PSM No Functional Vulnerability (N = 15,574)	*p* Value	SMD	After PSM Functional Vulnerability (N = 2720)	After PSM No Functional Vulnerability (N = 2720)	*p* Value	SMD
Age, years	76.8 ± 7.1	73.4 ± 6.8	<0.001	0.489	76.5 ± 7.0	76.3 ± 7.2	0.294	0.028
Female	1654 (58.1%)	8421 (54.1%)	<0.001	0.081	1568 (57.6%)	1552 (57.1%)	0.660	0.012
White	2086 (73.3%)	11,120 (71.4%)	0.041	0.042	1982 (72.9%)	1967 (72.3%)	0.653	0.013
Black	314 (11.0%)	1892 (12.1%)	0.091	0.034	306 (11.3%)	318 (11.7%)	0.613	0.013
Hispanic	256 (9.0%)	1504 (9.7%)	0.242	0.024	249 (9.2%)	255 (9.4%)	0.795	0.008
BMI, kg/m^2^	23.9 ± 5.2	25.6 ± 5.8	<0.001	0.308	24.1 ± 5.3	24.2 ± 5.4	0.486	0.019
Hypertension	1982 (69.6%)	9670 (62.1%)	<0.001	0.159	1870 (68.8%)	1856 (68.2%)	0.672	0.012
Type 2 diabetes	746 (26.2%)	3314 (21.3%)	<0.001	0.116	698 (25.7%)	686 (25.2%)	0.693	0.010
CKD	1021 (35.9%)	3904 (25.1%)	<0.001	0.236	952 (35.0%)	938 (34.5%)	0.684	0.011
CAD	802 (28.2%)	3596 (23.1%)	<0.001	0.117	758 (27.9%)	747 (27.5%)	0.743	0.009
Heart failure	621 (21.8%)	2112 (13.6%)	<0.001	0.216	578 (21.3%)	566 (20.8%)	0.673	0.011
COPD	704 (24.7%)	2956 (19.0%)	<0.001	0.138	668 (24.6%)	655 (24.1%)	0.667	0.011
Dementia	318 (11.2%)	842 (5.4%)	<0.001	0.212	285 (10.5%)	276 (10.1%)	0.691	0.010
Anemia	1596 (56.1%)	6428 (41.3%)	<0.001	0.300	1486 (54.6%)	1472 (54.1%)	0.706	0.011
Malnutrition/cachexia	724 (25.4%)	1682 (10.8%)	<0.001	0.385	642 (23.6%)	625 (23.0%)	0.591	0.015
Prior bowel resection	1018 (35.8%)	4636 (29.8%)	<0.001	0.128	958 (35.2%)	945 (34.7%)	0.709	0.010
Prior intestinal obstruction	692 (24.3%)	2542 (16.3%)	<0.001	0.200	640 (23.5%)	626 (23.0%)	0.658	0.012
Stricturing phenotype proxy	628 (22.1%)	2744 (17.6%)	<0.001	0.112	594 (21.8%)	582 (21.4%)	0.688	0.011
Penetrating phenotype proxy	312 (11.0%)	1448 (9.3%)	0.007	0.056	298 (11.0%)	289 (10.6%)	0.672	0.010
Corticosteroid exposure	1244 (43.7%)	5496 (35.3%)	<0.001	0.172	1162 (42.7%)	1148 (42.2%)	0.711	0.011
Biologic exposure	1032 (36.3%)	5942 (38.2%)	0.058	0.039	1001 (36.8%)	1014 (37.3%)	0.707	0.010
Immunomodulator exposure	386 (13.6%)	2318 (14.9%)	0.069	0.038	372 (13.7%)	378 (13.9%)	0.804	0.006
Hemoglobin, g/dL	10.6 ± 1.8	11.4 ± 1.9	<0.001	0.432	10.7 ± 1.8	10.8 ± 1.8	0.128	0.056
Albumin, g/dL	3.12 ± 0.62	3.48 ± 0.58	<0.001	0.598	3.16 ± 0.61	3.18 ± 0.60	0.224	0.033
Creatinine, mg/dL	1.22 ± 0.56	1.08 ± 0.49	<0.001	0.266	1.20 ± 0.55	1.19 ± 0.54	0.501	0.018
CRP, mg/L	34.8 ± 28.6	29.4 ± 25.8	<0.001	0.198	33.6 ± 27.9	32.8 ± 27.5	0.286	0.029

Abbreviations: BMI = body mass index; CAD = coronary artery disease; CKD = chronic kidney disease; COPD = chronic obstructive pulmonary disease; CRP = C-reactive protein; PSM = propensity score matching; SMD = standardized mean difference. Continuous variables are presented as mean ± standard deviation and categorical variables as n (%).

**Table 2:** Relative risks and risk differences for clinical outcomes after propensity score matching.

Outcome	Timepoint	Functional Vulnerability (n/N)	No Functional Vulnerability (n/N)	RD (95% CI)	RR (95% CI)	*p* Value
All-cause mortality	7 days	42/2720	24/2720	0.007 (0.002 to 0.012)	1.75 (1.07–2.87)	0.024
All-cause mortality	30 days	116/2720	72/2720	0.016 (0.007 to 0.026)	1.61 (1.21–2.14)	0.001
All-cause mortality	90 days	204/2720	146/2720	0.021 (0.008 to 0.035)	1.40 (1.14–1.72)	0.001
Bowel surgery	30 days	238/2720	184/2720	0.020 (0.007 to 0.033)	1.29 (1.07–1.56)	0.006
Sepsis	30 days	276/2720	196/2720	0.029 (0.015 to 0.044)	1.41 (1.18–1.68)	<0.001
Acute kidney injury	30 days	402/2720	318/2720	0.031 (0.014 to 0.048)	1.26 (1.10–1.44)	<0.001
ICU admission	30 days	314/2720	238/2720	0.028 (0.013 to 0.043)	1.32 (1.13–1.55)	<0.001
Venous thromboembolism	30 days	68/2720	46/2720	0.008 (0.001 to 0.015)	1.48 (1.02–2.14)	0.036
TPN use	30 days	214/2720	146/2720	0.025 (0.013 to 0.037)	1.47 (1.20–1.79)	<0.001
Blood transfusion	30 days	348/2720	276/2720	0.026 (0.011 to 0.042)	1.26 (1.09–1.46)	0.002
All-cause readmission	30 days	486/2720	398/2720	0.032 (0.013 to 0.052)	1.22 (1.08–1.39)	0.001
All-cause readmission	90 days	754/2720	642/2720	0.041 (0.018 to 0.064)	1.17 (1.07–1.29)	<0.001

Abbreviations: CI = confidence interval; ICU = intensive care unit; RD = risk difference; RR = relative risk; TPN = total parenteral nutrition.

**Table 3:** Length of stay and selected continuous outcomes after propensity score matching.

Outcome	Functional Vulnerability (N = 2720)	No Functional Vulnerability (N = 2720)	Mean Difference (95% CI)	*p* Value
Hospital length of stay, days	8.9 ± 6.1	6.7 ± 5.2	2.2 (1.9 to 2.5)	<0.001
Peak creatinine, mg/dL	1.54 ± 0.83	1.36 ± 0.75	0.18 (0.14 to 0.22)	<0.001
Change in creatinine from baseline, mg/dL	0.34 ± 0.52	0.23 ± 0.46	0.11 (0.08 to 0.14)	<0.001
Hemoglobin nadir, g/dL	9.2 ± 1.7	9.6 ± 1.8	−0.4 (−0.5 to −0.3)	<0.001
Peak CRP, mg/L	52.6 ± 38.4	45.1 ± 35.8	7.5 (5.5 to 9.5)	<0.001

Abbreviations: CI = confidence interval; CRP = C-reactive protein.

**Table 4:** Cox proportional hazards models for outcomes associated with administratively defined functional vulnerability in the propensity-matched cohort.

Outcome	HR	Coefficient	SE	z	*p* Value	95% CI
90-day all-cause mortality	1.38	0.322	0.104	3.10	0.002	1.13–1.69
Bowel surgery	1.27	0.239	0.085	2.81	0.005	1.07–1.51
Sepsis	1.39	0.329	0.076	4.33	<0.001	1.20–1.61
Acute kidney injury	1.24	0.215	0.061	3.52	<0.001	1.10–1.40
ICU admission	1.31	0.270	0.071	3.80	<0.001	1.14–1.51
TPN use	1.43	0.358	0.085	4.21	<0.001	1.21–1.69
90-day readmission	1.18	0.166	0.044	3.77	<0.001	1.08–1.29

Abbreviations: CI = confidence interval; HR = hazard ratio; ICU = intensive care unit; SE = standard error; TPN = total parenteral nutrition.

**Table 5:** Sensitivity analyses using restrictive functional vulnerability definitions and exclusion of malnutrition/cachexia codes.

Analysis	Outcome	Functional Vulnerability Events	No Functional Vulnerability Events	RR (95% CI)	HR (95% CI)	*p* Value
≥2-code functional vulnerability definition	30-day mortality	62/1180	34/1180	1.82 (1.21–2.74)	1.70 (1.18–2.45)	0.004
≥2-code functional vulnerability definition	Sepsis	138/1180	86/1180	1.60 (1.24–2.06)	1.54 (1.22–1.95)	<0.001
≥2-code functional vulnerability definition	AKI	208/1180	154/1180	1.35 (1.12–1.62)	1.31 (1.11–1.55)	0.001
≥2-code functional vulnerability definition	90-day readmission	346/1180	292/1180	1.18 (1.04–1.34)	1.17 (1.04–1.31)	0.008
Excluding malnutrition/cachexia	30-day mortality	74/2030	48/2030	1.54 (1.08–2.19)	1.46 (1.07–1.99)	0.018
Excluding malnutrition/cachexia	Sepsis	188/2030	142/2030	1.32 (1.08–1.62)	1.29 (1.07–1.56)	0.007
Excluding malnutrition/cachexia	AKI	284/2030	232/2030	1.22 (1.04–1.42)	1.19 (1.04–1.37)	0.012
Excluding malnutrition/cachexia	90-day readmission	548/2030	480/2030	1.14 (1.03–1.26)	1.13 (1.03–1.24)	0.010

Abbreviations: AKI = acute kidney injury; CI = confidence interval; HR = hazard ratio; RR = relative risk.

**Table 6:** Subgroup analysis of 30-day mortality, bowel surgery, and sepsis after propensity score matching.

Subgroup	n Functional Vulnerability/No Functional Vulnerability	30-Day Mortality RR (95% CI)	*p* Value	Bowel Surgery RR (95% CI)	*p* Value	Sepsis RR (95% CI)	*p* Value
Stricturing phenotype proxy	594/582	1.55 (0.86–2.78)	0.141	1.34 (1.01–1.78)	0.041	1.48 (1.09–2.01)	0.012
Penetrating phenotype proxy	298/289	1.68 (0.77–3.67)	0.191	1.22 (0.84–1.78)	0.291	1.53 (1.01–2.32)	0.044
Prior bowel resection	958/945	1.44 (0.91–2.28)	0.114	1.31 (1.03–1.66)	0.028	1.37 (1.08–1.73)	0.009
No prior bowel resection	1762/1775	1.66 (1.14–2.42)	0.008	1.24 (0.97–1.59)	0.083	1.42 (1.14–1.77)	0.002
Biologic exposure	1001/1014	1.36 (0.86–2.15)	0.184	1.18 (0.91–1.53)	0.209	1.29 (0.99–1.68)	0.058
No biologic exposure	1719/1706	1.73 (1.19–2.50)	0.004	1.36 (1.09–1.70)	0.006	1.49 (1.22–1.82)	<0.001
Total matched cohort	2720/2720	1.61 (1.21–2.14)	0.001	1.29 (1.07–1.56)	0.006	1.41 (1.18–1.68)	<0.001
Interaction *p* value			0.744		0.812		0.693

Abbreviations: CI = confidence interval; RR = relative risk.

## Data Availability

The data that support the findings of this study are available from TriNetX, but restrictions apply to the availability of these data, which were used under license for the current study and are therefore not publicly available. Data may be available from the authors upon reasonable request and with permission from TriNetX.

## Abbreviations

AKI	Acute kidney injury
BMI	Body mass index
CAD	Coronary artery disease
CI	Confidence interval
CKD	Chronic kidney disease
COPD	Chronic obstructive pulmonary disease
CPT	Current Procedural Terminology
CRP	C-reactive protein
EHR	Electronic health record
HCO	Healthcare organization
HIPAA	Health Insurance Portability and Accountability Act
HR	Hazard ratio
IBD	Inflammatory bowel disease
ICD-10-CM	International Classification of Diseases, Tenth Revision, Clinical Modification
ICD-10-PCS	International Classification of Diseases, Tenth Revision, Procedure Coding System
ICU	Intensive care unit
PSM	Propensity score matching
RD	Risk difference
RR	Relative risk
SE	Standard error
SMD	Standardized mean difference
STROBE	Strengthening the Reporting of Observational Studies in Epidemiology
TPN	Total parenteral nutrition
VTE	Venous thromboembolism
